# Radiosensitivity and Capacity to Recover from Radiation-Induced Damage in Pimonidazole-Unlabeled Intratumor Quiescent Cells Depend on *p53* Status

**DOI:** 10.4021/wjon272w

**Published:** 2011-02-26

**Authors:** Shin-ichiro Masunaga, Yong Liu, Hiroki Tanaka, Yoshinori Sakurai, Minoru Suzuki, Natsuko Kondo, Akira Maruhashi, Koji Ono

**Affiliations:** a Particle Radiation Oncology Research Center, Research Reactor Institute, Kyoto University, 2-1010, Asashiro-nishi, Kumatori-cho, Sennan-gun, Osaka 590-0494, Japan; b Radiation Medical Physics, Research Reactor Institute, Kyoto University, 2-1010, Asashiro-nishi, Kumatori-cho, Sennan-gun, Osaka 590-0494, Japan

**Keywords:** Quiescent cell, Pimonidazole, Oxygenation status, *p53* status, Microenvironment, Gamma-rays

## Abstract

**Background:**

Using our method for selectively detecting the response of intratumor quiescent (Q) cells to irradiation, the Q cells was shown to have a much larger hypoxic fraction (HF) than total (= proliferating (P) + Q) tumor cell population irrespective of the *p53* status of tumor cells. However, the size of the HF was clearly less than 100%, meaning the Q cell population was never fully hypoxic. Thus, the dependency of the radio-sensitivity and recovery capacity from radiation-induced damage on *p53* status was investigated in pimonidazole-unlabeled oxygenated Q tumor cells.

**Methods:**

Human head and neck squamous cell carcinoma cells transfected with mutant *TP53* (SAS/mp53), or with neo vector as a control (SAS/neo), were inoculated subcutaneously into left hind legs of Balb/cA nude mice. The tumor-bearing mice received 5-bromo-2’-deoxyuridine (BrdU) continuously to label all intratumor P cells. Tumors were irradiated with γ-rays at a high dose-rate or a reduced dose-rate at 1 h after the administration of pimonidazole. The responses of Q and total cell populations were evaluated with the frequencies of micronucleation and apoptosis using immunofluorescence staining for BrdU. The response of pimonidazole unlabeled tumor cell fractions was assessed with apoptosis frequency using immunofluorescence staining for pimonidazole.

**Results:**

The pimonidazole-unlabeled tumor cell fraction showed significantly enhanced radio-sensitivity compared with the whole tumor cell fraction more remarkably in Q cells and *p53*-mutated tumors than total cells and *p53*-wild type tumors, respectively. However, a significantly greater decrease in radio-sensitivity in the pimonidazole-unlabeled than the whole cell fraction, evaluated using a delayed assay or a decrease in radiation dose rate, was more clearly observed in Q cells and *p53*-wild type tumors than total cells and *p53*-mutated type tumors, respectively. Concerning the whole tumor cell fraction, the Q cells showed significantly greater radio-resistance and recovery capacity from radiation-induced damage than the total cells both in *p53*-wild and *p53*-mutated type tumors.

**Conclusions:**

The pimonidazole-unlabeled sub-fraction of the Q tumor cells, probably oxygenated, may be a critical target in the control of solid tumors, although its radio-sensitivity and recovery capacity from radiation-induced damage depend on *p53* status of the tumor cell.

## Introduction

Human solid tumors are thought to contain moderately large fractions of quiescent (Q) tumor cells, which are not involved in the cell cycle and have stopped dividing, but which are as viable as established experimental animal tumor lines [1]. The presence of Q cells is probably due, at least in part, to hypoxia and the depletion of nutrition in the tumor core as a consequence of poor vascular supply [1]. As a result, with the exception of non-viable Q cells at the very edge of the necrotic rim where there is diffusion-limited hypoxia, Q cells are viable and clonogenic but have ceased dividing.

Using our method for selectively detecting the response of Q cells in solid tumors to treatment that damages DNA, the Q cell population in solid tumors has been shown to exhibit more resistance to conventional radio- and chemotherapy. The Q cell population has also been demonstrated to have more capacity to recover from radiation- and chemotherapeutic agent-induced damage and to have a significantly larger hypoxic fraction (HF) irrespective of the *p53* status of tumor cells. However, the Q cell population in solid tumors has never been shown to be fully hypoxic [2]. Actually, the size of the HF of the Q cell population in the SCC VII squamous cell carcinomas tumor, implanted in the hind legs of C3H/He mice and with a diameter of 1 cm, were 55.1 ± 6.2 (mean ± SE) % [3]. Thus, the size of the HF in a Q cell population was significantly less than 100%, indicating that the Q cell population undoubtedly includes oxygenated tumor cells.

A few years ago, the universal detection of hypoxic cells in both tissues and cell cultures became possible using pimonidazole, a substituted 2-nitroimidazole, and a mouse IgG1 monoclonal antibody (MAb1) to stable covalent adducts formed through reductive activation of pimonidazole in hypoxic cells [4]. Here, we tried to selectively detect the response of the pimonidazole non-labeled and probably oxygenated cell fraction of the Q cell population. To achieve this we combined our method for selectively detecting the response of Q cells in solid tumors with the method for detecting cell and tissue hypoxia using pimonidazole and MAb1 to pimonidazole.

It has been shown that the *p53* tumor suppressor gene serves a critical role in maintaining genomic stability during a cell cycle checkpoint in not only G1 but also the G2/M transition [5], as an effector of DNA repair and apoptosis. *p53* is mutated in a majority of human solid tumors and plays a central role in the cellular response to DNA damaging treatments like ionizing radiation, chemotherapy, or hypoxic stress [6]. Hypoxic stress also induces p53 protein accumulation and p53-dependent apoptosis, but does not induce p53-dependent cell cycle arrest [6]. Loss of *p53* function may result in resistance to DNA-damaging agents, including ionizing radiation and hypoxic stress [7]. Thus, the genetic and functional status of the *p53* gene is thought to be an important factor in guiding therapeutic strategies for cancer patients.

Intensity modulated radiotherapy and stereotactic irradiation have become common as new radiotherapy modalities for the treatment of malignancies. These techniques often require precise positioning of patients and longer exposure times in a single treatment session [8, 9]. Prolongation of irradiation time may induce adverse radiation effects and evokes major concern related to the dose-rate effect. Thus, there is a need to clarify the effect of a reduction in dose-rate on the radio-sensitivity of tumors *in vivo*.

In the present study, the radio-sensitivity of the pimonidazole non-labeled cell fraction of the Q cell populations within *p53* wild- and mutated-type tumors, following γ-ray irradiation at both a high dose rate (HDR) and a reduced dose rate (RDR), was determined. This is almost the first attempt to evaluate the response of oxygenated fractions of Q tumor cells *in vivo*, referring to its dependency on *p53* status using two different solid tumors identical in genetic background except for *p53* status.

## Materials and Methods

### Cells, tumors, and mice

The human head and neck squamous cell carcinoma cell line SAS (provided by JCRB, Tokyo) was cultured at 37 *°*C in Dulbeco’s Modified Eagle’s medium (DMEM) containing 20 mM 2-[4-(2-hydroxyethyl)-1-piperazinyl] ethanesulfonic acid (HEPES) and 12.5% fetal bovine serum in a conventional humified 5% CO_2_ incubator. Although SAS cells have a homozygous point mutation (GAG to TAG) at codon 336 of exon 10 in the oligomerization domain of an *mp53* gene, they show the phenotype of wild-type p53 in radiation- and heat-induced signal transduction [10]. SAS/mp53 and SAS/neo cells were obtained by the transfection of dominant-negative p53 (Arg248Trp) expressing plasmid, pC53-248, and control empty plasmid pCMV-Neo-Bam (provided by B. Vogelstein, Johns Hopkins Oncology Center, Baltimore, MD). As a result, SAS/neo cells have a functional p53 protein, and SAS/mp53 cells express a dominant negative p53 protein. The procedure is described in detail elsewhere [11].

Cells were collected from exponentially growing cultures, and 5.0 x 10^5^ cells were injected subcutaneously into the left hind legs of 6 - 7 weeks old syngeneic female Balb/cA nude mice. Three weeks after inoculation, a tumor with a diameter of approximately 7 mm could be observed at each implanted site, whichever stable transfectant was used. At this size, no necrotic component was observed within the both SAS/neo and SAS/mp53 tumors. The body weight of the tumor-bearing mice was 20.1 ± 2.3 (Mean ± standard error) g. Mice were handled according to the Recommendations for Handling of Laboratory Animals for Biomedical Research, compiled by the Committee on Safety Handling Regulations for Laboratory Animal Experiments, Kyoto University.

### Labeling with 5-bromo-2’-deoxyuridine (BrdU)

Two weeks after tumor cell inoculation, mini-osmotic pumps (Alzet model 2001, DURECT Corporation, Cupertino, CA) containing BrdU dissolved in physiological saline (250 mg/ml) were implanted subcutaneously to label all proliferating (P) cells for 7 days. Administration of BrdU did not change the tumor growth rate. The tumors were approximately 7 mm in diameter on treatment. The percentage of labeled cells after continuous labeling with BrdU was 48.4 ± 6.7% and 43.2 ± 6.2% for SAS/neo and SAS/mp53 tumor cells, respectively, and reached a plateau level at these stages. Therefore, we regarded tumor cells not incorporating BrdU after continuous labeling as Q cells.

### Treatment

After labeling with BrdU, tumor-bearing mice received an intraperitoneal administration of pimonidazole hydrochloride (Hypoxyprobe Inc., Burlington, MA, USA) dissolved in physiological saline at a dose of 60 mg/kg. Ninety minutes later, mice were irradiated with γ-rays without anesthesia. Individual animals were secured in a specially designed device made of acrylic resin with the tail firmly fixed with adhesive tape. γ-rays were delivered using a cobalt-60 γ-ray irradiator at dose rates of 2.5 and 0.039 Gy/min representing a HDR and a RDR irradiation, respectively.

Each treatment group also included mice that had not been pretreated with BrdU.

### Immunofluorescence staining of BrdU-labeled and/or pimonidazole-labeled cells and the observation of apoptosis and micronucleation

Based on our previous report related to the determination of the timing of apoptosis [3], an apoptosis assay undertaken at 6 h after irradiation and a micronucleus assay carried out immediately after irradiation were used as immediate assays. Tumors were excised from mice given BrdU, weighed, minced and trypsinized (0.05% trypsin and 0.02% ethylenediamine-tetraacetic acid (EDTA) in phosphate-buffered saline (PBS) at 37 °C for 20 min). Furthermore, as a delayed assay, tumors were also excised from mice given BrdU, weighed, minced and trypsinized at 30 h after irradiation for the apoptosis assay, and at 24 h after irradiation for the micronucleus assay. For the apoptosis assay, single cell suspensions were fixed without further treatment. For the micronucleus assay, tumor cell suspensions were incubated for 72 h in tissue culture dishes containing complete culture medium and 1.0 µg/ml of cytochalasin-B, to inhibit cytokinesis while allowing nuclear division. The cultures were then trypsinized and cell suspensions were fixed. For both assays, after the centrifugation of fixed cell suspensions, the cell pellet was resuspended with cold Carnoy’s fixative (ethanol : acetic acid = 3 : 1 in volume). The suspension was placed on a glass microscope slide and the sample was dried at room temperature. Slides were treated with 2 M hydrochloric acid for 60 min at room temperature to dissociate the histones and partially denature the DNA. They were then immersed in borax-borate buffer (pH 8.5) to neutralize the acid. BrdU-labeled tumor cells were detected using indirect immunofluorescence staining with a rat monoclonal anti-BrdU antibody (Abcam plc, Cambridge, UK) and a goat Alexa Fluor 488-conjugated anti-rat IgG antibody (Invitrogen Corp., Carlsbad, CA, USA). Pimonidazole-labeled tumor cells were detected using indirect immunofluorescence staining with a mouse monoclonal anti-pimonidazole antibody (Hypoxyprobe Inc., Burlington, MA, USA) and a rabbit Alexa Fluor 594-conjugated anti-mouse IgG antibody (Invitrogen Corp., Carlsbad, CA, USA). To enable the observation of the triple staining of tumor cells with green-emitting Alexa Fluor 488 and red-emitting Alexa Fluor 594, cells on the slides were treated with blue-emitting 4’6-diamidino-2-phenylindole (DAPI) (0.5 µg/ml in PBS) and monitored under a fluorescence microscope.

The frequency of apoptosis in BrdU-unlabeled cells (= Q cells at irradiation) and pimonidazole-unlabeled tumor cells was determined by counting apoptotic cells in tumor cells that did not show green fluorescence from Alexa Fluor 488 and red fluorescence from Alexa Fluor 594, respectively. The apoptosis frequency was defined as the ratio of the number of apoptotic cells to the total number of observed tumor cells [3]. The micronucleus frequency in BrdU-unlabeled cells was examined by counting the micronuclei in the binuclear cells that did not show green fluorescence emitted by Alexa Fluor 488. The micronucleus frequency was defined as the ratio of the number of micronuclei in the binuclear cells to the total number of binuclear cells observed [2].

The ratios obtained in tumors not pretreated with BrdU indicated the apoptosis frequency and the micronucleus frequency in the total (P + Q) tumor cell populations. More than 300 tumor cells and binuclear cells were counted to determine the apoptosis frequency and the micronucleus frequency, respectively.

### Clonogenic cell survival assay

The clonogenic cell survival assay was also performed in the mice that were given no BrdU or pimonidazole using an *in vivo-in vitro* assay method. Tumors were disaggregated by stirring for 20 min at 37 °C in PBS containing 0.05% trypsin and 0.02% EDTA. The cell yield was (1.5 ± 0.3) x 10^7^/g and (3.4 ± 0.8) x 10^6^/g for SAS/neo and SAS/mp53 tumors, respectively. A colony formation assay using the *in vivo-in vitro* assay method was performed with the culture medium.

The apoptosis and micronucleus frequencies and surviving fractions for the total cell population were obtained from cells in tumors that were not pretreated with BrdU or pimonidazole. The apoptosis and micronucleus frequencies for Q cells were obtained from unlabeled tumor cells after continuous BrdU labeling without pimonidazole loading. The apoptosis frequencies for the total tumor cell population that were not labeled with pimonidazole were obtained from tumor cells that were not labeled with pimonidazole after pimonidazole loading without BrdU pretreatment. The apoptosis frequencies for Q cells that were not labeled with pimonidazole were obtained from tumor cells that were not labeled with BrdU or pimonidazole after both continuous BrdU labeling and pimonidazole loading [12]. Thus, there was no effect of interaction between BrdU and irradiation or between pimonidazole and irradiation on the values for the apoptosis and micronucleus frequencies and surviving fractions. Incidentally, since the rate of pimonidazole-labeled tumor cells could change during culturing with cytochalasin-B over 3 days, following the production of single tumor cell suspensions by excising and mincing the tumors from mice that underwent pimonidazole loading, the micronucleus frequency for the cell fraction that was not labeled with pimonidazole after pimonidazole loading was not determined. As a consequence, the radio-sensitivity of the pimonidazole unlabeled cell fractions was only determined in relation to apoptosis induction.

More than three tumor-bearing mice were used to assess each set of conditions and each experiment was repeated at least twice. To examine the differences between pairs of values, Student’s *t*-test was used when variances of the two groups were assumed to be equal; otherwise the Welch *t*-test was used.

## Results

The plating efficiency and the micronucleus and apoptosis frequencies after a radiation dose of 0 Gy are shown in Table 1. Overall, SAS/neo showed significantly higher plating efficiency and apoptosis frequency, and significantly lower micronucleus frequency than SAS/mp53 in both total and Q cell populations. The micronucleus and apoptosis frequencies were significantly higher for the Q cell population than for the total cell population in both SAS/neo and SAS/mp53 tumors. In contrast, the apoptosis frequency was significantly lower for the cell fraction that was not labeled with pimonidazole than for the whole tumor cell fraction in both total and Q cell populations in both tumors.

**Table 1:** Plating Efficiency and Micronucleus Frequency at 0 Gy

	Total tumor cells	Quiescent cells
**SAS/neo**		
Plating efficiency (%)	45.5 ± 8.9^a)^	
Micronucleus frequency	0.038 ± 0.006	0.056 ± 0.007
Apoptosis frequency	0.083 ± 0.008	0.106 ± 0.015
Especially in pimonidazole unlabeled cell fraction	0.045 ± 0.005	0.058 ± 0.008
**SAS/mp53**		
Plating efficiency (%)	23.5 ± 4.1	
Micronucleus frequency	0.072 ± 0.008	0.111 ± 0.010
Apoptosis frequency	0.021 ± 0.003	0.033 ± 0.004
Especially in pimonidazole unlabeled cell fraction	0.011 ± 0.001	0.019 ± 0.003

a): Mean ± standard error (n = 9)

**Figure 1:**
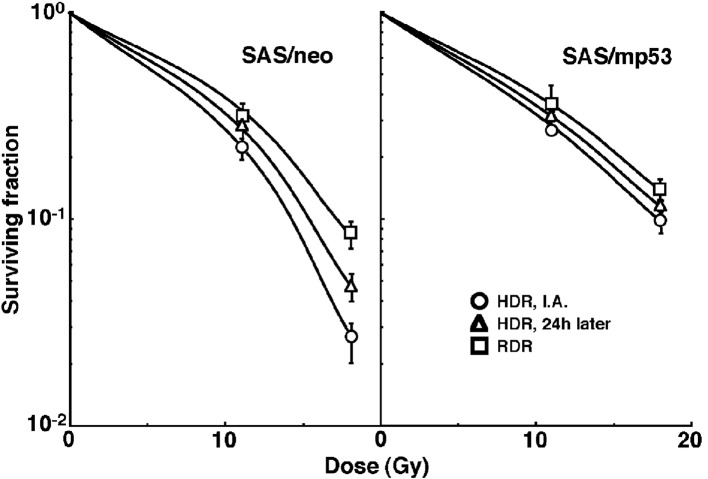
Cell survival curves for the whole tumor cell fraction in the total tumor cell population of SAS/neo (left panel) and SAS/mp53 (right panel) tumors as a function of radiation dose. Circles, triangles and squares represent the surviving fractions immediately after (I.A.), at 24 h after (24h later) high dose-rate (HDR), and reduced dose-rate (RDR) γ-ray irradiation, respectively. Bars represent standard errors.

Cell survival curves for the total tumor cell population as a function of radiation dose are shown in Figure 1. On the whole, SAS/mp53 showed the higher surviving fractions (SFs) than SAS/neo especially for higher radiation dose. The surviving fractions (SFs) increased in the following order: immediately after HDR irradiation < 24 h after HDR irradiation < immediately after RDR irradiation in both tumors.

**Figure 2:**
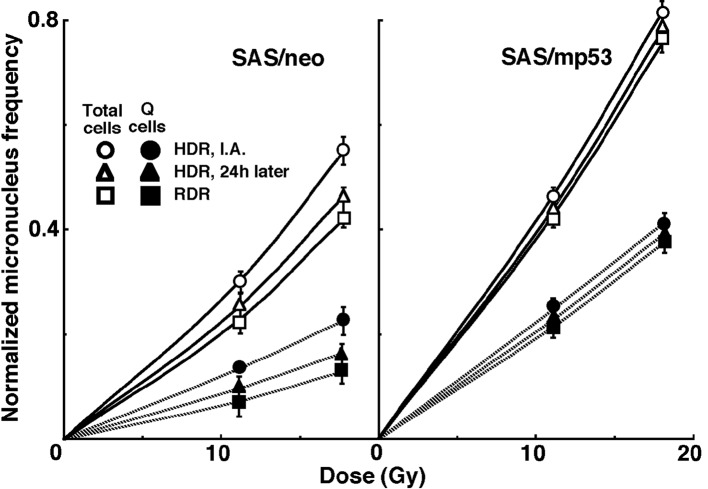
Dose response curves of the normalized micronucleus frequency for the whole tumor cell fraction in the total and quiescent (Q) tumor cell populations of SAS/neo (left panel) and SAS/mp53 (right panel) tumors as a function of radiation dose. Open and solid symbols represent the normalized micronucleus frequencies for total and quiescent tumor cell populations, respectively. Circles, triangles and squares represent the normalized micronucleus frequency immediately after (I.A.), at 24 h after (24h later) high dose-rate (HDR), and reduced dose-rate (RDR) γ-ray irradiation, respectively. Bars represent standard errors.

For baseline correction, we used the normalized micronucleus frequency to exclude the micronucleus frequency in non-irradiated tumors. The normalized micronucleus frequency was defined as the micronucleus frequency in the irradiated tumors minus the micronucleus frequency in the non-irradiated tumors. Dose response curves for the normalized micronucleus frequency in total and Q tumor cell populations as a function of radiation dose are shown in Figure 2. Overall, the normalized micronucleus frequencies were significantly lower in SAS/neo and the Q cells than in SAS/mp53 and the total cell population, respectively. In both the total and Q cell populations of the both tumors, the normalized micronucleus frequencies decreased in the following order: immediately after HDR irradiation > 24 hours after HDR irradiation > immediately after RDR irradiation.

**Figure 3:**
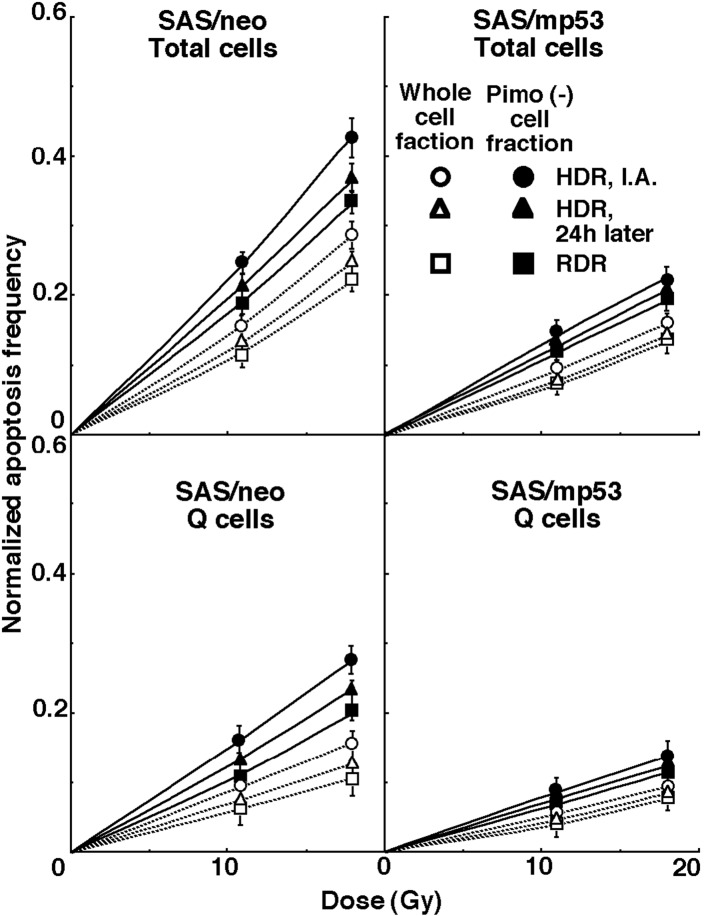
Dose response curves for the normalized apoptosis frequency of the total and quiescent (Q) tumor cell populations of SAS/neo (left panel) and SAS/mp53 (right panel) tumors as a function of radiation dose are shown in the upper and lower panels, respectively. Open and solid symbols represent the normalized apoptosis frequencies for the whole tumor cell fraction and the cell fraction not labeled with pimonidazole (Pimo (-)) in both the total and Q tumor cell populations, respectively. Circles, triangles and squares represent the normalized apoptosis frequency immediately after (I.A.), at 24 h after (24h later) high dose-rate (HDR), and reduced dose-rate (RDR) γ-ray irradiation, respectively. Bars represent standard errors.

For another baseline correction, we used the normalized apoptosis frequency to exclude the apoptosis frequency in non-irradiated tumors. The normalized apoptosis frequency was the apoptosis frequency in the irradiated tumors minus that in the non-irradiated tumors. Dose response curves for the normalized apoptosis frequency in the total and Q tumor cell populations as a function of radiation dose are shown in Figure 3. Overall, the normalized apoptosis frequencies were significantly lower in SAS/mp53 and the Q cells than in SAS/neo and the total cell population, respectively. Moreover, the normalized apoptosis frequency was significantly higher for the cell fraction that was not labeled with pimonidazole than for the whole tumor cell fraction in both the Q and total cell populations of the both tumors under each set of conditions. For both the pimonidazole unlabeled and the whole cell fractions, in both the Q and total tumor cell populations, the normalized apoptosis frequencies decreased in the following order: immediately after HDR irradiation > 24 h after HDR irradiation > immediately after RDR irradiation.

To evaluate the radio-sensitivity of the cell fraction that was not labeled with pimonidazole, as compared with the whole cell fraction in both the total and Q cell populations, dose-modifying factors (DMFs) were calculated using the data obtained under all irradiation conditions in Figure 3 (Table 2). The values for the DMFs were significantly higher than 1.0, and SAS/mp53 showed slightly higher values than SAS/neo under all sets of conditions. Overall, DMF values tended to be higher for the Q cell than total cell population, and in particular immediately after HDR irradiation. In the total cell population, the DMF values were almost constant. However, for Q cells, the DMF values had a tendency to decrease in the following order: immediately after HDR irradiation > 24 h after HDR irradiation > immediately after RDR irradiation.

**Table 2:** Dose-Modifying Factors for the Pimonidazole-Unlabeled Cell Fraction Compared with the All Cell Fraction in the Total or Quiescent Cell Population^a)^

	High dose-rate Immediately after	High dose-rate 24 hours after	Reduced dose-rate
**SAS/neo**			
Total cell population	1.45 ± 0.15^b)^	1.45 ± 0.15	1.45 ± 0.15
Quiescent cell population	1.6 ± 0.2	1.55 ± 0.15	1.55 ± 0.15
**SAS/mp53**			
Total cell population	1.5 ± 0.15	1.5 ± 0.15	1.5 ± 0.15
Quiescent cell population	1.8 ± 0.25	1.75 ± 0.2	1.7 ± 0.2

a): The ratio of the dose of radiation necessary to obtain the normalized apoptosis frequency of 0.05 in a whole cell fraction to that needed to obtain the frequency in the pimonidazole unlabeled cell fraction.

b): Mean ± standard error (n = 9)

To investigate the reduction in radio-sensitivity caused by a delayed assay or a decrease in the radiation dose rate, DMFs were calculated using the data for all irradiation conditions given in Figures through 1-3 (Table 3). Overall, SAS/mp53 showed lower DMF values than SAS/neo under all sets of conditions. On the whole, in the fraction unlabeled with pimonidazole or the whole cell fraction, the values were higher after RDR irradiation than at 24 h after HDR irradiation in both the total and Q cell populations, particularly in the latter population. The DMF values were significantly higher in the Q cell than total cell population in both the pimonidazole unlabeled and whole cell fractions. In both the Q and total cell populations, the values for pimonidazole unlabeled cell fractions were higher than those for the whole cell fractions, particularly in the case of the Q cells.

**Table 3:** Dose-Modifying Factors due to a Delayed Assay or Reduced Irradiation Dose-Rate^a)^

	High dose-rate 24 hours after	Reduced dose-rate
**SAS/neo**		
Surviving fraction = 0.2		
Total cells	1.1 ± 0.05^b)^	1.2 ± 0.1
Normalized micronucleus frequency = 0.1		
Total cells	1.15 ± 0.05	1.25 ± 0.1
Quiescent cells	1.3 ± 0.1	1.6 ± 0.2
Normalized apoptosis frequency = 0.05		
Total cells	1.15 ± 0.05	1.25 ± 0.1
Especially in pimonidazole unlabeled cell fraction	1.15 ± 0.05	1.3 ± 0.1
Quiescent cells	1.2 ± 0.1	1.4 ± 0.15
Especially in pimonidazole unlabeled cell fraction	1.35 ± 0.15	1.6 ± 0.2
**SAS/mp53**		
Surviving fraction = 0.2		
Total cells	1.05 ± 0.05	1.1 ± 0.05
Normalized micronucleus frequency = 0.1		
Total cells	1.05 ± 0.05	1.1 ± 0.05
Quiescent cells	1.1 ± 0.05	1.15 ± 0.05
Normalized apoptosis frequency = 0.05>		
Total cells	1.1 ± 0.05	1.15 ± 0.05
Especially in pimonidazole unlabeled cell fraction	1.15 ± 0.05	1.2 ± 0.1
Quiescent cells	1.15 ± 0.05	1.2 ± 0.1
Especially in pimonidazole unlabeled cell fraction	1.25 ± 0.1	1.3 ± 0.1

a): The ratio of the dose of radiation necessary to obtain each end-point with a delayed assay or reduced dose-rate irradiation to that needed to obtain each end-point with an assay immediately after high dose-rate irradiation.

b): Mean ± standard error (n = 9)

To examine the difference in radio-sensitivity between the total and Q cell populations, DMFs that allow us to compare the dose of radiation necessary to obtain each end-point in the two cell populations were calculated using the data in Figure 2 and 3 (Table 4). All DMF values were significantly higher than 1.0, and SAS/neo showed slightly higher values than SAS/mp53 under each set of condition (Table 4). The DMF values increased in the following order: immediately after HDR irradiation < 24 h after HDR irradiation < immediately after RDR irradiation. The values were lower for the sub-population that was not labeled with pimonidazole as compared with the whole cell population in the both tumors.

**Table 4:** Dose-Modifying Factors for Quiescent Cell Population Relative to Total Tumor Cell Population^a)^

	High dose-rate Immediately after	High dose-rate 24 hours after	Reduced dose-rate
**SAS/neo**			
Normalized micronucleus frequency = 0.1	2.0 ± 0.2^b)^	2.35 ± 0.2	2.65 ± 0.25
Normalized apoptosis frequency = 0.05	1.7 ± 0.15	1.75 ± 0.2	1.85 ± 0.2
Especially in pimonidazole unlabeled cell fraction	1.55 ± 0.1	1.6 ± 0.15	1.65 ± 0.15
**SAS/mp53**			
Normalized micronucleus frequency = 0.1	1.8 ± 0.2	1.8 ± 0.2	1.85 ± 0.2
Normalized apoptosis frequency = 0.05	1.65 ± 0.15	1.7 ± 0.15	1.75 ± 0.2
Especially in pimonidazole unlabeled cell fraction	1.5 ± 0.1	1.55 ± 0.1	1.6 ± 0.15

a): The ratio of the dose of radiation necessary to obtain each end-point in the quiescent cell population to that needed to obtain each end-point in the total tumor cell population.

b): Mean ± standard error (n = 9)

## Discussion

In recent years, the concept of cancer stem cells (CSCs) or tumor-initiating cells (tumor clonogens) has attracted a great deal of interest because of the potential clinical significance [13]. In part, these cells are thought to exist in a pathophysiological microenvironment where hypoxia, low pH and nutrient deprivation occur. Further, under these microenvironmental conditions, dividing tumor cells have also been thought to become quiescent. Actually, a subset of CSCs or tumor clonogens consists of non-dividing quiescent cells [14]. Thus, in the present study we tried to clarify the radiobiological characteristics of the sub-population in the intratumor Q cell population in the context of CSC or tumor clonogen characteristices.

The fraction of cells that were not labeled with pimonidazole showed significantly higher radio-sensitivity than the whole cell fraction in both the Q and total cell populations, and amongst the Q cells and within the SAS/mp53 tumor in particular (Table 2). This was probably because pimonidazole unlabeled cells were more oxygenated than the whole cell fraction, which comprised both oxygenated and hypoxic tumor cells, in both the Q and total tumor cell populations [4]. Additionally, the Q cell population and the SAS/mp53 tumor as a whole included a larger HF than the total tumor cell population and the SAS/neo tumor, respectively [15]. As shown in Table 3, the pimonidazole unlabeled cell fraction had a greater recovery capacity than the whole cell fraction, especially in the case of the Q cells and within the SAS/neo tumor. As reported previously [16], the SAS/neo tumor showed much greater recovery capacity than the SAS/mp53 tumor and radio-sensitivity decreased in the following order: immediately after HDR irradiation, at 24 h after HDR irradiation, and immediately after RDR irradiation, particularly in the Q cells (Table 3). As a consequence, in the case of the Q cells, the difference in radio-sensitivity between the pimonidazole-unlabeled and whole cell fractions declined in the same order (Table 2). One mechanism of CSC or tumor clonogen resistance to cytotoxic treatment is supposed to be based on an enhanced DNA repair capacity [17]. Here, the pimonidazole-unlabeled Q cell fraction showed a much greater recovery capacity than the Q cell population as a whole, even if the recovery capacity was significantly greater in the entire Q cell population than in the total tumor cell population as a whole. In other words, from the viewpoint of not only quiescent status but also enhanced DNA repair capacity, the characteristics of the pimonidazole-unlabeled cell fraction in the Q cell population were found to be similar to those of CSCs or tumor clonogens.

In SAS/mp53 solid tumors, cells with mutant *p53* could escape from the checkpoint mechanism for maintaining genomic stability by wild-type p53, and proliferate without cell cycle arrest or apoptosis [6]. Thus, as is often the case with advanced malignant solid tumors, in due course, neovascularization could not catch up with the rapid tumor cell proliferation, resulting in a large HF and Q cell fraction in solid tumors. Now, p53 status is supposed to have the potential to influence microenvironmental conditions, such as the cellularity and the size of the HF in solid tumors [1, 15].

The microenvironmental conditions under which dividing tumor cells become quiescent might promote the formation of micronuclei and apoptosis at 0 Gy in the whole Q tumor cell fractions, partly due to hypoxic stress (Table 1) [1]. In the current study, the Q cells were shown to be significantly less radiosensitive and to have a greater recovery capacity than the total cell population (Fig. 2, 3 and Table 3, 4). This finding indicated that more Q cells survive radiation therapy than P cells. In particular, in the cell fraction that was not labeled with pimonidazole, the difference in radio-sensitivity between the Q and total cell populations was markedly increased when evaluated using the delayed assay or by employing a reduced radiation dose-rate. This was due to the greater recovery capacity of the unlabeled Q cell fraction as compared with the unlabeled cell fraction in the total tumor cell population (Table 4). Thus, whether the pimonidazole-unlabeled or the whole Q cell population, control of the Q cells could have a great impact on the outcome of radiation therapy and the Q cell population can be a critical target in the control of solid tumors.

Improving the oxygenation status in cultured tumor cells and solid tumors following irradiation has been shown to promote recovery from radiation-induced damage in some previous reports [18, 19]. In the present study, the pimonidazole-unlabeled, and probably oxygenated, cell fraction showed a greater recovery capacity than the Q cell population as a whole. This finding was also observed more or less in both *p53* wild- and mutated-type tumors. However, although there is a similarity between the pimonidazole-unlabeled Q cell fraction and CSCs or tumor clonogens in terms of quiescent status and enhanced recovery capacity, CSCs or tumor clonogens are thought to exist under rather hypoxic conditions [14]. In the future, using human tumor cell lines, the characteristics of the intratumor Q cell population in connection with those of CSCs or tumor clonogens also have to be analyzed.
